# Glances at the Hospitals

**Published:** 1904-10-15

**Authors:** 


### GLANCES AT THE HOSPITALS.

### COVENTRY AND WARWICKSHIRE HOSPITAL.

The total ordinary income amounted to £4,229, and the total ordinary expenditure to £4,919. This shows a deficit of £690. It is satisfactory to note that the workmen's Saturday Fund amounted to £1,725, and the whole of the manufacturing firms in the neighbourhood are being approached to obtain their promise to contribute to the hospital a similar sum to that of their respective work-people. This is an excellent idea, and we hope it will be found successful. We see that a bazaar produced £3,617, a cycle parade £75, and an annual ball £41. The extraordinary income for the year reached £4,917, and the expenditure was £670. The accommodation in the present hospital is inadequate to the necessities of the district and it is proposed to add considerably to the 72 beds which it at present contains. The operating theatre is out of date, and this will also have to be dealt with. The hospital contained 66 patients at the beginning of the year, and 777 were admitted during it, making a total of 843 under treatment. Of these 616 were discharged cured, 83 relieved, at own request 12, 63 died, and 69 remained in the hospital. The above numbers yield the following percentages: cured 79·5 per cent., relieved 10·7 per cent., and died 8·2 per cent. The daily average number resident was 64. The average cost per bed occupied was £66 8s., the average cost per patient was £5 9s., and the cost per day was 3s. 7⅝d. The cost of the out-patients is estimated at 2s. each. The medical and surgical tables are quite useless for comparison with other hospitals, and there is neither an operation table nor an anæsthetic table.

### THE SOUTHAMPTON HOSPITAL.

The total ordinary income amounted to £5,607, and the total ordinary expenditure to £9,086, showing an excess of expenditure over income of £3,479. But as a set-off against this we may note that legacies to the amount of £11,668 were received. Still it is not on the uncertain amount of legacies that a hospital must rely for its income; and the committee appeal most earnestly to all supporters to try to enlist the help of others, and this appeal is more than justifiable seeing that the district served by the hospital has a population of 300,000, and that the annual subscriptions only amounted to £1,749. The donations and subscriptions of the workmen themselves were only £128, a sum so absurdly small that it ought to be at least quadrupled. The hospital exists almost solely to aid these working people when they are sick, and how can they expect the inhabitants of South Hants and Southampton to come forward when they do so little for themselves. The daily average number resident was 106, and the number of patients admitted during the year was 1,553. The cost per bed occupied was £73 3s.; the cost per patient was £4 19s.; and the cost per patient per day was almost 4s. Of the total number of patients 905 were discharged cured, 288 were discharged relieved, 108 for various reasons, 79 died, and 103 remained under treatment. An analysis of the medical and surgical in-patients is given, but as no results are stated, the statistics are useless. A table of surgical operations is printed, and we notice that of 4 cases of nephrolithotomy 2 were cured and 2 relieved. Of 3 cases of nephrectomy 1 was cured, 1 relieved, and 1 died. Of 7 cases of ovariotomy 4 were cured, 2 relieved and one died. Altogether 1,305 operations were performed, but unfortunately no anæsthetic table is given.

### THE ADELAIDE CHILDREN'S HOSPITAL.

The income was £6,214, and the expenditure exceeded this by £17; but of the gross receipts £315 was placed to the endowment fund, so that the financial position of the hospital is sound. The institution contains a Roentgen Rays department and a bacteriological department, and both of these are extensively used not only for the Children's hospital but for medical men in private practice; in fact, the fees obtained therefrom have more than met the working expenses. Improvements have been carried out during the year, and additions are to be made to the laundry and to the accommodation for nurses. At the beginning of the year the hospital contained 68 patients, and 636 were admitted during it, making a total of 704 under treatment. Of these, 428 were cured, 130 relieved, 50 discharged not relieved, 34 died, and 62 remained in the wards. The tables show the numbers of patients treated, the numbers "cured," "relieved," "not relieved," and "died." There were 78 cases of diphtheria with 3 deaths, 50 cases of enteric fever without a death, and 20 cases of rheumatic fever with 1 death. These results are very satisfactory. So with 31 cases of pneumonia and 4 deaths. The total number of operations performed was 466, of which 102 were on in-patients, and 364 on out-patients. The anæsthetic table shows that chloroform was the agent used in 50 cases, ether in 58, gas in 4, chloroform followed by ether in 353, and gas followed by ether in 1.

